# The complete genome of two *Lactiplantibacillus plantarum* isolates from the traditional Mexican fermented pulque beverage assembled with a combination of PacBio and Illumina platforms

**DOI:** 10.1128/mra.00985-23

**Published:** 2023-12-19

**Authors:** Elisa Karen Gutiérrez Rodríguez, Fernando Astudillo-Melgar, Violeta Larios, Francisco Bolívar, Adelfo Escalante, Martha Giles-Gómez

**Affiliations:** 1 Departamento de Biología, Facultad de Química, Universidad Nacional Autónoma de México, Ciudad Universitaria, Coyoacán, Ciudad de México, México; 2 Departamento de Ingeniería Celular y Biocatálisis, Instituto de Biotecnología, Universidad Nacional Autónoma de México, Cuernavaca, Morelos, México; 3 WinterGenomics, Ciudad de México, México; University of Southern California, Los Angeles, California, USA

**Keywords:** pulque, *Lactiplantibacillus plantarum*, complete genome, PacBio, Illumina NovaSec

## Abstract

We report the sequence of the complete genome and associated plasmids of two *Lactiplantibacillus plantarum* isolates from the traditional Mexican pulque beverage assembled with a combination of PacBio and Illumina data. The resulting complete genome for strain LB1_P46 is 3,287,706 bp; for strain LB2_P47, the complete genome is 3,289,072 bp.

## ANNOUNCEMENT

The traditional Mexican alcoholic beverage pulque is a source of potential probiotic bacteria (1 – 4). We report the complete sequences of the genome and the associated plasmids of two *Lactiplantibacillus plantarum* isolated from pulque with *in vitro* and *in vivo* probiotic properties (5), sequenced by Pacific Biosciences (PacBio) and Illumina NovaSeq platforms.

The studied bacteria were isolated from pulque sampled from Huitzilac, Morelos (19°01′42″N 99°16′02″W), Mexico, according to ISO 20128:2006 (6). Colonies were purified by the streak-plate method. Gram staining and catalase-negative tests were applied. Isolated strains were maintained in glycerol stocks at −70°C. According to the supplier’s directions, DNA was extracted from an overnight culture in De Man, Rogosa, and Sharp (MRS) broth at 30°C without shaking with the DNeasy UltraClean Microbial Kit (Qiagen, Hilden, Germany). The quality of the DNA was assessed on a 1% agarose gel. Ten micrograms of DNA of each isolate extracted from an overnight culture was sent to Novogene Co., Ltd (Sacramento, CA, USA) for library construction, quality control, and sequencing by NovaSeq PE150 and PacBio Sequel II/IIe systems according to Novogene Co., Ltd.

For the NovaSeq platform, libraries were constructed with the NEBNext Ultra^T^ II DNA Library Prep Kit (NE BioLabs, Ipswich, MA, USA). For PacBio, DNA samples were fragmented and size-selected using the BluePippin system (Sage Science) to select a size of 13 kb. DNA fragments were damaged, repaired, end-repaired, and A-tailed. The SMRTbell template prep kit v2.0 (PacBio) was used for library preparation. After the exonuclease and AMPure PB beads’ purification steps, the sequencing primer was annealed to the SMRTbell templates, followed by the binding of the sequencing polymerase to the annealed templates. In both procedures, libraries were checked with Qubit for quantification and with a bioanalyzer for size distribution detection. Quantified libraries were pooled and sequenced.

PacBio and NovaSeq data quality control was performed with FastQC (v0.12.1) (https://www.bioinformatics.babraham.ac.uk/projects/fastqc). The summary of the raw data results of PacBio and NovaSeq is shown in Table 1. The PacBio .bam files were converted to .fastq using the program Bam2Fastx v1.0.01 (https://github.com/PacificBiosciences/pbbioconda), and assembling was performed with Unicycler v0.4.8 tool (7), assembling bacterial genomes from a combination of short and long reads. The assembly of NovaSeq and PacBio sequencing data resulted in four contigs for LB1_P46 and LP2 in five contigs. We analyzed the resulting contigs in the BLAST v.2.11.0 program (8) to determine if they matched with genomes or plasmids in the non-redundant database. The larger contigs corresponded to the closed genomes, and the smaller corresponded to closed plasmids. Table 1 shows the assemble summary of genomes and plasmids. Identity of the larger assembled contigs determined by BLAST resulted in assigning the identification of two *Lactiplatibacillus plantarum* (query cover 100%, identity 99.80%). Gene annotation for chromosomes and plasmids was performed by the NCBI Prokaryotic Genome Annotation Pipeline (9).

**TABLE 1:** Sequence features and accession number of assembled replicons of *Lactiplantibacillus plantarum* strains isolated from pulque

Strain	Raw data			SRA number		Total assembly length (bp)	Coverage	Total no. CDS	GC content (%)	Contig/replicon	GenBank accession number	Length (bp)
	PacBio		NovaSeq	PacBio	NovaSeq							
	Read number	N50 read length	Raw reads									
LB1_P46	29,636	161,519	8,577,280	SRX22037557	SRX22037556	3,287,706	195.63×	3,027	44	Contig_1/Closed Chromosome	CP135983.1	3,189,825
										Contig_2/Closed plasmid 1	CP135984.1	47,688
										Contig_3/Closed plasmid 2	CP135985.1	38,244
										Contig_4/Closed plasmid 3	CP135986.1	11,949
LB2_P47	26,674	161,519	9,406,070	SRX22037559	SRX22037558	3,289,072	214.46×	3,029	43	Contig_1/Closed Chromosome	CP135978.1	3,189,831
										Contig_2/Closed plasmid 1	CP135979.1	38,244
										Contig_3/Closed plasmid 2	CP135980.1	28,315
										Contig_4/Closed plasmid 3	CP135981.1	20,733
										Contig_5/Closed plasmid 4	CP135982.1	11,949

## Data Availability

The assembled sequences of *Lactiplantibacillus plantarum* isolates LB1_P46 and LB2_P47 were deposited in the GenBank database under BioProject PRJNA1021245, Biosample SAMN37547333 (LB1_P46), and Biosample SAMN37547334 (LB2_P47). SRA numbers for each strain are shown in Table 1.
